# Tocilizumab and Abatacept for the Treatment of Childhood Chronic Uveitis: A Monocentric Comparison Experience

**DOI:** 10.3389/fped.2022.851453

**Published:** 2022-04-12

**Authors:** Ilaria Maccora, Sarah Abu Rumeileh, Franco Curci, Cinzia de Libero, Edoardo Marrani, Maria Vincenza Mastrolia, Ilaria Pagnini, Gabriele Simonini

**Affiliations:** ^1^Rheumatology Unit, Meyer Children's University Hospital, Florence, Italy; ^2^NeuroFARBA Department, University of Florence, Florence, Italy; ^3^Ophthalmology Unit, Meyer Children's University Hospital, Florence, Italy

**Keywords:** uveitis, children, JIA, Behçet, Tocilizumab, Abatacept, biologics

## Abstract

**Background:**

Our study aimed to evaluate the efficacy of Tocilizumab and Abatacept for treating Childhood Chronic non-infectious Uveitis (CCU), resistant to anti-tumor necrosis factor (anti-TNF) treatment.

**Methods:**

This is a monocentric retrospective charts review study (January 2010–April 2021) recruiting CCU, refractory to anti-TNF. To be included, children should have active uveitis at the time of Tocilizumab (8 mg/kg, every 4 weeks) or Abatacept (10 mg/kg, every 4 weeks). The main outcome was the achievement of ocular remission on treatment defined as the absence of flares for ≥ 6 months.

**Results:**

In this study, 18 patients with CCU (14 F), previously treated with Methotrexate and Adalimumab, were enrolled: 15 had juvenile idiopathic arthritis (JIA) (83.3%), 2 idiopathic (11.1%), and 1 Behçet (5.6%). Furthermore, ten patients received Abatacept and 8 patients received Tocilizumab. The mean duration of treatment on Abatacept was 31.6 months (SD ± 30.8), on Tocilizumab 25.25 months (SD ± 17.8). In total, 13 children (72.2%) achieved remission, with a better remission rate for the Tocilizumab group (8/8) compared to the Abatacept group (5/10) (χ^2^ 5.53, *p* = 0.019). No difference was evaluated between the two groups in the proportion of patients who showed flares during the treatment (2/6 Abatacept vs. 1/8 Tocilizumab). A significant difference was evaluated in the proportion of patients who flared after treatment discontinuation: 3/3 Abatacept vs. 0/3 Tocilizumab (χ^2^ 3.8, *p* = 0.025).

**Conclusion:**

Even though this is a monocentric retrospective study, in a relatively small group, our study suggests a superior efficacy of Tocilizumab over Abatacept for treating anti-TNF refractory CCU.

## Introduction

Childhood chronic non-infectious uveitis (CCU), a rare, but sight-threatening disease, may require early and aggressive treatment to avoid several complications, including blindness (1–3). Even though it may be idiopathic in up to 50% of cases, it may be associated with different systemic diseases as juvenile idiopathic arthritis (JIA), in about 40% of cases, or Behçet syndrome (2–5).

In the last two decades, the prognosis has been dramatically changed thanks to the increasing knowledge about the physiopathology of the disease and the use of new drugs that target the specific molecules of inflammation as the tumor necrosis factor—TNFα (1).

Two recent randomized controlled trials (RCTs) showed the efficacy of Adalimumab in the treatment of anterior CCU, refractory to common Disease Modifying Anti-Rheumatic Drugs (DMARDs) (6, 7). Ramanan et al. (6) showed that Adalimumab decreased the hazard of treatment failure by 75% and treatment failure was only observed in the 27% of patients treated with this drug, while Quartier (7) showed that 56.5% of patients had a decreased rate of inflammation.

A recent meta-analysis highlighted the role of Adalimumab, over Infliximab, as an effective treatment in CCU, displaying a proportion of favorable outcomes at 86% (95% *CI*: 76–95%) (8).

However, when a patient failed this treatment, there is a lack of evidence regarding the best available approach (1, 9–12). Current recommendations did not suggest one drug over another, based on the existing knowledge (3, 10, 11).

Up to now, several case series of CCU have been published about the efficacy of Tocilizumab, an anti-IL6 inhibitor, and Abatacept, a CTLA-4 antagonist (13–21).

A recent trial of phase II investigating the efficacy of Tocilizumab in JIA-associated uveitis has been published. Although it did not achieve the primary end point, 33% of patients had a two-step decrease in ocular inflammation and 75% of patients had a complete resolution of macular edema (22). Conversely, the result of the trial about the use of Abatacept in childhood uveitis is still awaiting publishing in a journal (NCT01279954).

To date, no data from comparative studies have been published about the efficacy and safety of Tocilizumab and Abatacept for the treatment of CCU.

This study aimed to compare the efficacy and safety of Tocilizumab and Abatacept in the treatment of pediatric patients with chronic non-infectious uveitis resistant to Adalimumab in a monocentric retrospective cohort.

## Methods

### Study Design, Setting, Population

This was a retrospective, non-interventional, monocentric, comparative cohort study involving the Rheumatology and Ophthalmology Units of Meyer Children's University Hospital. Patients of these units have been included in the present study if they attended the clinics between January 2010 and April 2021 and fulfilled the following inclusion criteria: (i) the diagnosis of CCU according to the definition of chronic uveitis of Standardization of Uveitis Nomenclature Working Group (23), (ii) diagnosis previous than 16 years-old, (iii) to be refractory at least to a common Disease Modifying anti-Rheumatic Drugs (DMARDs) and at least a first course of anti-TNF, (iv) to be treated with intravenously Tocilizumab (8 mg/kg every 4 weeks) or intravenously Abatacept (10 mg/kg every 4 weeks), (v) age under 18 years old during treatment, and (vi) to have a follow-up of at least 6 months with the treatment on study.

We considered the following exclusion criteria: (i) to start the drug in a study due to the concomitant systemic disease status rather than to control the uveitis activity, and (ii) to have a demyelinating disease.

The study was carried out in accordance with the Helsinki adherence to the tenets of the Declaration of Helsinki and the ethic committee of Meyer Children's University Hospital approved the study.

### Data Collections

Data were retrieved by the revision of the medical records of patients with CCU treated with Tocilizumab or Abatacept and collected in an *ad hoc* Excel customized database.

For each patient, the following information has been collected: demographic data (gender, date of birth, age at onset of the disease, and age when the drugs on studies were started), patient history (personal and familial history), characteristics of the disease (type of disease: idiopathic, JIA-associated uveitis, Behçet; laterality and anatomical site of uveitis), laboratory data at onset (positivity for ANA, ANCA, HLA B27, HLA B51, erythro-sedimentation rate (ESR) expressed in mm/h, C-reactive protein (CRP) expressed in mg/dl), previous systemic treatment performed before Tocilizumab and/or Abatacept.

To evaluate the efficacy of Tocilizumab and Abatacept, the medical records of patients with CCU were retrieved to collect, at the time of the drugs starting and then every 3 ± 1 months, the following data: anterior chamber cells and flare grading according to SUN, bio score, the presence of active retinal vasculitis, visual acuity reported in logMAR, and when this scale was not available the appropriate conversion will be performed according to Schulze-Bonsel et al. (24), the presence and number of complications, type of complications as cataract, increased intraocular pressure (>21 mmHg), ocular hypotony (<5 mmHg), optic disc swelling, macular edema or thickness (defined as increased thickness in the macula on OCT scan), posterior synechiae, epiretinal membrane, band keratopathy, retinal vasculitis, multifocal choroiditis, choroidal neovascular membrane, surgical treatment, data on concomitant therapies (topical corticosteroid drops, administration of systemic corticosteroids, and concomitant DMARDs) and, regarding safety data, the number of adverse events (AEs) and any severe AEs with their description. A serious AE was defined as an event secondary to a drug exposition that leads to death, life-threatening events, events conducive to prolonging hospitalization, enduring or significant disability/ incapacity, or medical events needing medical or surgical intervention to prevent a serious outcome or congenital anomaly/birth defect (25).

Visual acuity has been stratified as normal if LogMAR was <0.3, impaired if 0.3–1, and blindness if >1.

### Main Outcomes Measures

The main outcome was the achievement of ocular remission on treatment defined as the inactive disease for ≥6 consecutive months, receiving systemic therapy. The inactive disease was defined as less than or equal to 0.5+ anterior chamber cells, less than or equal to 0.5+BIO score/National Eye Institute (NEI) vitreous haze scale, no active retinal or choroidal lesions, after discontinued any steroid treatment, including topical treatment, and no declaration of treatment failure due to intolerability or safety concerns (26). Treatment failure was defined as failure to reduce eye drops to 2 drops/day by or at the 12 week visit, the development of new complications, or intolerance/non-adherence treatment (23).

To compare their potential long-lasting effect on maintaining remission, primary outcomes once the remission on medication was achieved were as follows: (a) the rate of patients who relapse after disease remission and (b) the time to the first relapse on treatment. Additional secondary outcomes, once the drug in the study was started were as follows: (a) time to achieve remission, (b) the rate of relapse when the drug is discontinued, and (c) the time to the first relapse after the drug in the study was discontinued.

### Statistical Analysis

Statistical analyses were performed with SPSS27.0 for Windows. Continuous variables were reported as median and range, while categorical variables as frequencies and percentages. Mann–Whitney *U*-test, Wilcoxon's signed rank test for paired samples, chi-square tests, and Fisher's exact test, when appropriate, were used to compare data. The following data, entered into a customized uveitis database, were considered as covariates for the survival curves, age at the initiation of/age at the initiation of therapies in studies, gender, associated autoimmune disease, disease duration, uveitis duration, the interval between the uveitis onset and the initiation of the drug in the study, concomitant medications, previous corticosteroid use, previous disease modifying anti-rheumatic drug treatment duration, number of patients with eye complications due to chronic uveitis (including glaucoma, synechiae, band keratopathy, cystoid macular edema, vitreitis, and cataract), and follow-up time. To identify the predictors of outcome, Kaplan–Meier curves were constructed, each one at the mean of the covariates reported above. For each subject, the total number of AEs and serious ADEs was calculated. A *p* < 0.05 was considered significative.

## Results

In total, 18 patients were enrolled in the study (14 women, 77.8%), with a median age at onset of the disease of 29 months (range 12–105 months) with a median follow-up of 22.5 months (range 3–97 months). Of 18 patients, fifteen had JIA-associated uveitis (83.3%), 2 idiopathic uveitis (11.1%), and 1 Behçet syndrome (5.6%). In 16 patients, the uveitis was anterior (88.9%), in 2 posterior (11.1%), and 11 had a bilateral involvement (61.1%). ANA positivity was recorded in 15 patients (83.3%), while ANCA in 4 (22.2%). Demographic and clinical characteristics are summarized in Table 1. Demographic parameters, laboratory data, and other reported variables in the statistical analysis section did not differ between the 2 groups.

**Table 1:** The demographic and clinical characteristics of population in study.

**Variable**	**Entire cohort**	**TOC**	**ABA**	**Differences between TOC vs. ABA**
	**(18 patients)**	**(8 patients, 44.4%)**	**(10 patients, 55.6%)**	***P***
Gender, *n* of female (%)	14 F/18 (77.8%)	6 F/8 (75%)	8F/10 (80%)	NS
Age at onset months [median (range)]	29	30	29	NS
	(12–105)	(12–105)	(20–83)	
**Disease [*****n*** **(%)]**				
JIA-U	15 (83.3%)	6 (75%)	9 (90%)	NS
Behçet	1 (5.6%)	1 (12.5%)	0	
Idiopathic	2 (11.1%)	1 (12.5%)	1 (10%)	
Presence of comorbidity [*n* (%)]	2	1	1	NS
	(11.1%)	(12.5%)	(10%)	
ANA positivity [*n* (%)]	15 (83.3%)	6 (75%)	9 (90%)	NS
ANCA positivity [*n* (%)] (data in 12/18)	4 (22.2%)	2 (25%)	2 (20%)	NS
HLA B51	1 (5.6%)	1 (12.5%)	0	NS
ESR mm/h (median)	41 (4–92)	35.5 (21.4)	48.2 (SD 25.2)	NS
CRP mg/dl (median)	0 (0)	1 (SD 0)	1 (SD 0)	NS
Laterality of uveitis		6 (75%)	5 (50%)	NS
Bilateral [*n* (%)]	11 (61.1%)			
**Anatomical location of uveitis**				
Anterior	16 (88.9%)	7 (87.5%)	9 (90%)	NS
Intermediate	0			NS
Posterior	2 (11.1%)	1 (12.5%)	1 (10%)	
Panuveitis	0			
**Previous treatments**				
MTX [*n* (%)]	18 (100%)	8 (100%)	10 (100%)	NS
ETA [*n* (%)]	4 (22.2%)	2 (25%)	2 (20%)	NS
ADA [*n* (%)]	18 (100%)	8 (100%)	10 (100%)	NS
IFX [*n* (%)]	1 (5.6%)	–	1 (10%)	NS
GOL [*n* (%)]	1 (5.6%)	1 (12.5%)	–	NS
CAN [*n* (%)]	1 (5.6%)	1 (12.5%)	–	NS
ABA [*n* (%)]	3 (16.7%)	3 (37.5%)	–	NS

*ABA, abatacept; TOC, Tocilizumab; F, female; ANA, antinuclear antibody; ESR, erythro-sedimentation rate; CRP, C-reactive protein; NS, not significative; JIA-U, juvenile idiopathic associated uveitis; n, number; SD, standard deviation; MTX, methotrexate; ETA, etanercept; ADA, adalimumab; CAN, canakinumab; Gol, golimumab; IFX, infliximab*.

Ten patients received Abatacept (55.6%) while 8 patients received Tocilizumab (44.4%). Three of the 8 patients treated with Tocilizumab, previously received Abatacept and discontinued it for treatment failure.

All the patients included in the study were previously treated with Methotrexate and Adalimumab before switching to Tocilizumab or Abatacept. Over the disease course, due to active arthritis, 4 JIA children have received Etanercept before uveitis onset, and 2 additional children received Infliximab and Golimumab, respectively. The child with Behçet received Canakinumab before uveitis became the major complication.

The median time before Abatacept was administered from the onset of the uveitis was 49.5 months (range 12–127), while for Tocilizumab was 67 months (range 29–156) without significative differences. The median duration of the two treatments was 23 months (range 7–97) and 18 months (range 9–66) for Abatacept and Tocilizumab, respectively, without significative differences between the two groups.

Among the 18 patients, 11 were treated with Methotrexate as concomitant therapy (61.1%), while one was treated with Azathioprine (5.6%). When the two drugs were started, 5/10 and 4/8 patients also received systemic corticosteroid, respectively, in the Abatacept group and Tocilizumab group. Moreover, patients treated with Tocilizumab received a mean of 2.13 (SD ±1.8) drops of topical corticosteroids, while those treated with Abatacept received 2 (SD ±1.5).

When the drugs in the study have been started, a significative higher proportion of patients treated with Tocilizumab had complications compared to those treated with Abatacept (3/8 vs. 0/10, χ^2^ 4.09 *p* = 0.04) with an increased number of complications (*p* < 0.0001). Table 2 reported the characteristics of the eyes when the 2 drugs were started and at the last available follow-up.

**Table 2:** The characteristics of eyes in the two groups of treatment at the baseline and at the last available follow-up on treatment.

	**Variables**	**Toc (8 patients)**	**Aba (10 patients)**	**Toc vs. Aba**
Onset	Median age at the moment of the drug administration	118.5 (65–191)	113.5 (35–151)	0.28
	Median time from disease onset and first administration (range)	67 (29–156)	49.5 (12–127)	0.45
	Duration of therapy median (range)	18 (9–66)	23 (7–97)	0.61
	No. of corticosteroid drops [mean (SD)]	2.13 (1.8)	2 (1.5)	0.3
	No. of patients in systemic corticosteroid	4	5	0.81
	Visual acuity LogMar [mean (SD)]	0	0	
	Patients with complications (*n*)	3	0	**0.043**
	No. of complications mean (SD)	0.5 (0.756)	0 (0)	**<0.001**
	Posterior synechiae (*n*)	1	0	0.27
	Optic disc swelling (*n*)	3	0	0.11
	Choroid neovascular membrane (*n*)	1	0	0.27
Last FU	No. of drops of corticosteroid [mean (SD)]	0.25 (0.7)	0.56 (1.33)	0.28
	No. of patients in systemic corticosteroid	0	2	0.15
	Visual acuity [mean (SD)]	0.018 (0.05)	0.0	**0.035**
	Patients with complications (*n*)	2	2	0.8
	N° of Complications [mean (SD)]	0.25 (0.4)	0.33 (0.7)	0.42
	Posterior synechiae (*n*)	1	1	0.9
	Optic disc swelling (*n*)	1	0	0.25
	Choroid neovascular membrane (*n*)	1	0	0.25
	Band Keratopathy (*n*)	1	0	0.38

*Bold values are significative results*.

At the last available follow-up on therapy of the whole cohort (median 18 months, range 7–97 months), none of the patients treated with Tocilizumab and 2 of those with Abatacept were, respectively, on therapy with systemic corticosteroid. At that time, 2 patients for each group had complications, with no significative difference in the mean number of complications between the two groups (*p* 0.42). Optic disc swelling, reported in 3/8 patients when Tocilizumab was started, was completely resolved in 2/3 at the last available follow-up. No difference was evaluated in the mean visual acuity at the onset of the drugs between the two groups and it was in the normal range. However, at the last available follow-up, a significative difference was evaluated in the mean visual acuity (0.018 in Tocilizumab vs. 0 in Abatacept, *p* < 0.035). Nevertheless, all the patients had normal visual acuity.

Thirteen children (72.2%) achieved remission, with a better remission rate for Tocilizumab (8/8, 100%) compared to Abatacept (5/10, 50%) (χ^2^ 5.53, *p* = 0.019). The mean time to achieve remission with Tocilizumab was 10.88 months (SD ± 4.39), while with Abatacept was 11.8 months (SD ± 3.4) without significative difference. At 66 months of follow-up, which was the longest period common to the 2 groups, no significative difference was evaluated between the two groups in the proportion of patients who flared over the treatment: 2/5 with Abatacept and 1/8 with Tocilizumab (χ^2^ 1.3, *p* < 0.25), respectively, at 22.5 ± 9.19 months (mean ± SD) and at 11 months after starting the treatment.

Five patients discontinued the treatment due to persistent ocular remission: 3 were on Abatacept, at a median time of 40 months (range 35–53 months); 2 were on Tocilizumab, at a median time of 31 months (range 16–66 months). A significant difference was evaluated between the two groups in the proportion of patients who flared after treatment discontinuation: 3/3 with Abatacept and 0/2 with Tocilizumab (χ^2^ 3.8, *p* < 0.025) (Table 3).

**Table 3:** Main outcomes and adverse events.

**Variables**	**Tocilizumab**	**Abatacept**	**Toc vs. Aba**
	**(8 patients)**	**(10 patients)**	***P***
Achievement of initial response	8	8	0.18
Time to achieve response to therapy	3.63 (2.66)	3.63 (2.82)	1
Achievement of remission	8 (100%)	5 (50%)	**0.019**
Time to achieve remission	10.88 (4.39)	11.8 (3.4)	0.3
Flare on therapy	1	2	0.25
Time to first flare on therapy	–	22.5 (9.19)	0.07
Flare out of therapy	0/2	3/3	**0.025**
Time to first flare out of therapy	–	11 (5.5)	
**Adverse events**			
Patients that experience AEs [*n* (%)]	5 (71.4%)	2 (28.6%)	0.06
Patients that experience SAEs [*n* (%)]	0	0	–
No. of AEs [mean (SD)]	1 (1.06)	0.2 (0.42)	0.091
Hematological AEs	3 (neutropenia)	0	0.034
Gastrointestinal AEs	0	1 (persistent diarrhea)	0.35
Hepatobiliary AEs	1	0	0.25
Infections	1	0	0.25
Dermatological AEs	0	1	

*Bold values are significative results*.

**Figure 1:**
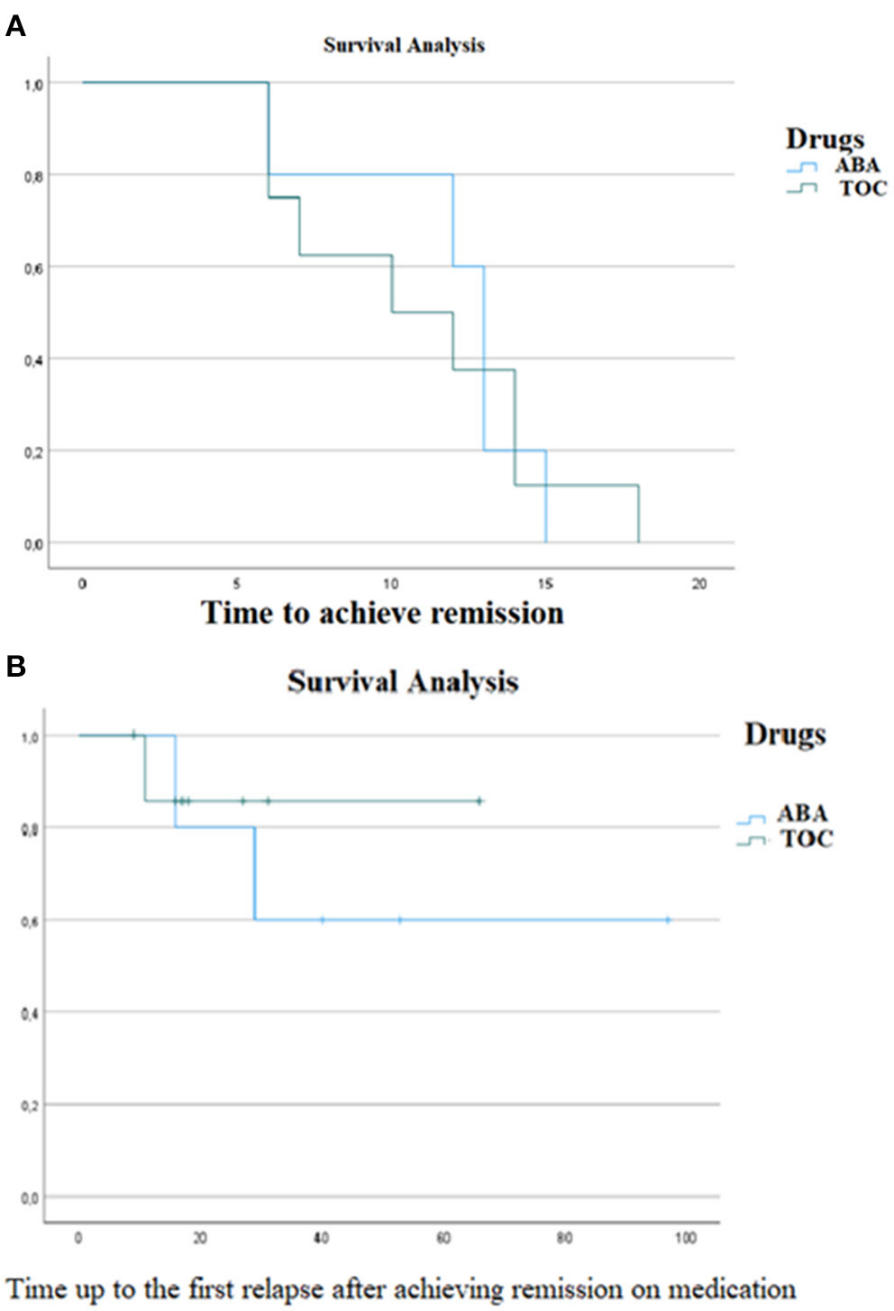
A survival analysis was conducted to evaluate difference **(A)** in the time to achieve remission and **(B)** in the time to first relapse after remission was achieved, between the group of patients treated with Tocilizumab and the group of patients treated with Abatacept. No significative difference were evaluated in the two outcomes [**(A)** χ^2^ 0, *p* 0.999; **(B)** χ^2^0.29, *p* 0.5]. ABA, Abatacept; TCZ, Tocilizumab.

At the mean of the above-mentioned covariates, including the total length of follow-up time of the 2 cohorts, the survival analysis did not show any difference between the two groups in terms of time to achieve remission on medication and time to the first relapse after remission was achieved on medication (Figures 1A,B).

Regarding drug safety over the period of observation, 5 patients experienced at least one AE with Tocilizumab, while 2 with Abatacept. None of the patients showed serious AEs. Among the patients treated with Tocilizumab, 3 patients experienced mild neutropenia, 1 experienced mildly increased transaminase, and 1 experienced upper airway infection with a concomitant rash. One additional patient with JIA discontinued Tocilizumab at 9 months due to persistent joint activity, nonetheless a persistent ocular remission. One patient discontinued Abatacept after 3 months of treatment, due to persistent diarrhea, and the patient was non-considered as not responder according to our main outcome measure. One additional patient on Abatacept experienced persistent dermatitis, later disappeared, however, keeping the treatment (Table 3).

## Discussion

Even if limited to a relatively small group, this comparative monocentric cohort study suggests that Tocilizumab is more efficacious than Abatacept in a mean period of treatment of 22.5 months in anti-TNF refractory CCU, with regard to the achieved remission on medication and maintaining remission after the discontinuation of treatment.

The two drugs in this peculiar setting showed significative differences in the proportion of patients who achieved remission, but no significative difference in the term of time to achieve remission, rate of relapse on therapy, and time to the first relapse on therapy.

To our knowledge, no studies or randomized clinical trials comparing Tocilizumab vs. Abatacept have been published to date for pediatric patients. Nonetheless, when patients failed to achieve disease control with Adalimumab, it is a key decision to find the appropriate drug that is able to control the inflammation and prevent ocular damages and visual loss.

The results of several case series are in accordance with our results about the proportion of responding children. Previous groups reported a rate of inactivity with Abatacept of 54% (17/35) and 52% (11/21), respectively (18, 20); although Tappeiner et al. (20) showed that uveitis recurred in 8/11 of patients who achieved inactivity, a higher percentage compared to our results. Regarding Tocilizumab, it has been reported inactive disease in 10/17 (58.8%) of patients (16) and improvement after 1 year of treatment in 15/17 patients (88.2%), with a complete remission in 19/25 (79%) of patients (13). These data are in accordance with ours and showed a higher proportion of children responding to Tocilizumab compared to Abatacept.

Moreover, Tocilizumab seems to be a valid option in patients who have failed Abatacept as demonstrated in our cohort of patients and in those of Calvo-Rio et al. (13), containing 3/8 and 6/25 patients previously treated with that drug, respectively. Moreover, in a recent revision of the literature, Cunningham et al. (27) highlighted the potential use of Tocilizumab to treat ocular inflammatory disease refractory to the conventional immunosuppressive therapies, including TNF inhibitors, as second or third-line therapy, not only in adult patients but also in children. However, literature results showed that when Tocilizumab has been administered subcutaneously, it showed a decrease in activity in controlling ocular inflammation and maintaining remission (22, 28).

According to recent data (17), children with optic disc swelling have been treated with Tocilizumab and showed a significant improvement and a decreased number of complications. No child with optic swelling has been treated with Abatacept. Therefore, a comparative analysis for this item had not been performed. However, even though this is an inherent selection bias, this datum mirrors the efficacy of Tocilizumab in treating macula edema/optic disc swelling of CCU (12, 15, 21). Moreover, in our cohort we observed a reduction of complications in patients treated with Tocilizumab and an increase of them in those treated with Abatacept at the last available follow-up, eliminating the differences highlighted at the beginning of the drugs.

To our knowledge, our study is the first to show an increased proportion of patients who relapsed after Abatacept withdrawal compared to Tocilizumab. Keeping in mind the limited number of included patients, if this datum will be duplicated in a larger, multicenter cohort, it would be an added value in the favor of Tocilizumab over Abatacept for this clinical setting.

As already identified (13, 17, 18, 22) as a significant outcome in childhood, we confirmed the corticosteroid-sparing effect, systemic and topical, of Tocilizumab and Abatacept, albeit to a lesser extent for this latter.

Both drugs evaluated in our study have proven to be safe in this peculiar real-life setting, and no serious adverse events occurred during the follow-up. Only one patient stopped Abatacept for persistent diarrhea as AE.

Before drawing our conclusions, several caveats need to be discussed.

We acknowledge that the retrospective study design, the small sample size, and the heterogeneous sample cohort with regard to the underlying disease (JIA, Behçet disease, and idiopathic uveitis) and the concomitant therapy (Methotrexate, Azathioprine, and systemic corticosteroid) results in significant bias that may hamper the comparison of the treatment outcomes of the two different cohorts. However, due to the rarity of the disease (anti-TNF refractory childhood chronic uveitis), the small number of eligible subjects meant that we could not consider results separately according to the underlying disease or different concomitant therapies. In addition, since we did not find statistically significant differences with regard to the demographic and clinical characteristics of the population in the study (Table 1), the 2 groups may be considered homogenous and thus comparable.

The retrospective nature of the study is an additional caveat that limits the interpretation of the present results. We recognize randomized controlled trials as the gold standard in comparing the disease outcomes between the two drugs: overall, a prospective study results in a valuable and superior approach. Therefore, we acknowledge the generally poor quality of evidence. Anyway, this is a monocentric study reporting real-life data in routine clinical care over a long-term follow-up. In our practice, standardized outcome measures according to current literature are routinely used and have been previously used over the entire study period. The use of Kaplan–Meyer curve analysis with the use of the same outcome measures over time are corrective procedures that are able to balance the potential bias related to the variable “TIME,” including specific bias associated with the retrospective nature of the study design. Taking together these considerations and the complete lack of evidence regarding this topic, a comparative analysis between two arms may be feasible and a retrospective design appears reasonable. Anyway, as a matter of fact, a prospective multicenter comparative study might be the answer to the clinical dilemma of what drug has to be chosen in childhood chronic uveitis after the anti-TNF α failure.

In conclusion, even though this is a monocentric retrospective study, in a relatively small group, our study seems to suggest a superior efficacy of Tocilizumab over Abatacept for treating anti-TNF refractory CCU. Additionally, Tocilizumab seems to be also effective in patients previously treated with Abatacept and has a steroid-sparing effect with no significant adverse events, irrespective of the underlying associated disease.

## Data Availability Statement

The raw data supporting the conclusions of this article will be made available by the authors, without undue reservation.

## Ethics Statement

The studies involving human participants were reviewed and approved by Meyer Children's Hospital. Written informed consent to participate in this study was provided by the participants' legal guardian/next of kin.

## Author Contributions

IM and GS contributed to the conception and design of the study and performed the statistical analysis. SA and FC organized the database. IM wrote the first draft of the manuscript. EM, IP, and MM wrote several sections of the manuscript. All authors contributed to manuscript revision, read, and approved the submitted version.

## Conflict of Interest

The authors declare that the research was conducted in the absence of any commercial or financial relationships that could be construed as a potential conflict of interest. The reviewer SG declared past co-authorships with one of the authors MM and GS and the absence of any ongoing collaboration with any of the authors to the handling editor.

## Publisher's Note

All claims expressed in this article are solely those of the authors and do not necessarily represent those of their affiliated organizations, or those of the publisher, the editors and the reviewers. Any product that may be evaluated in this article, or claim that may be made by its manufacturer, is not guaranteed or endorsed by the publisher.
